# Natalizumab Treatment Reduces Fatigue in Multiple Sclerosis. Results from the TYNERGY Trial; A Study in the Real Life Setting

**DOI:** 10.1371/journal.pone.0058643

**Published:** 2013-03-21

**Authors:** Anders Svenningsson, Eva Falk, Elisabeth G. Celius, Siegrid Fuchs, Karen Schreiber, Sara Berkö, Jennifer Sun, Iris-Katharina Penner, for the TYNERGY trial investigators

**Affiliations:** 1 Dept of Pharmacology and Clinical Neuroscience, Umeå University and University Hospital of Northern Sweden, Umeå, Sweden; 2 BiogenIdec Sweden AB, Upplands Väsby, Sweden; 3 Department of Neurology, Oslo University Hospital, Ullevål, Norway; 4 Department of Neurology, University of Graz, Graz, Austria; 5 Department of Neurology, Copenhagen University Hospital Rigshospitalet, Copenhagen, Denmark; 6 BiogenIdec Global Medical Affairs, Boston, Massachusetts, United States of America; 7 Department of Cognitive Psychology and Methodology, University of Basel, Basel, Switzerland; Charite Universitätsmedizin Berlin, Germany

## Abstract

Fatigue is a significant symptom in multiple sclerosis (MS) patients. First-generation disease modifying therapies (DMTs) are at best moderately effective to improve fatigue. Observations from small cohorts have indicated that natalizumab, an antibody targeting VLA-4, may reduce MS-related fatigue. The TYNERGY study aimed to further evaluate the effects of natalizumab treatment on MS-related fatigue. In this one-armed clinical trial including 195 MS patients, natalizumab was prescribed in a real-life setting, and a validated questionnaire, the Fatigue Scale for Motor and Cognitive functions (FSMC), was used both before and after 12 months of treatment to evaluate a possible change in the fatigue experienced by the patients. In the treated cohort all measured variables, that is, fatigue score, quality of life, sleepiness, depression, cognition, and disability progression were improved from baseline (all p values<0.0001). Walking speed as measured by the six-minute walk-test also increased at month 12 (p = 0.0016). All patients were aware of the nature of the treatment agent, and of the study outcomes.

**Conclusion:**

Natalizumab, as used in a real-life setting, might improve MS-related fatigue based on the results from this one-armed un-controlled stud. Also other parameters related to patients' quality of life seemed to improve with natalizumab treatment.

**Trial Registration:**

ClinicalTrials.gov NCT00884481

## Introduction

Fatigue is one of the major symptoms in multiple sclerosis (MS), affecting 54 to 95% of patients [1], [2], [3]. Fatigue is often present at disease onset, persists throughout the disease course, and negatively affects quality of life [4], [5]. So far, the pathophysiology is unknown, although there is strong evidence based on imaging studies that it may be of central origin. Fatigue seems to be closely related to the amount of atrophy [6], [7], [8], to lesions located predominantly in the frontal and parietotemporal white matter [6] and functional alterations in prefrontal cortex, thalamus and basal ganglia [9], [10]. The association of fatigue to clinical variables such as disease duration, relapse rate or disability is weak [1], [11], [12], making fatigue difficult to predict for individual patients.

In an attempt to capture the main features of MS-related fatigue, in 1998 an expert panel provided the following definition: “A subjective lack of physical and/or mental energy that is perceived by the individual or caregiver to interfere with usual and desired activities” [13]. Thus, to assess mental and physical fatigue in clinical practice, instruments are required which capture the whole spectrum of the symptom. The Fatigue Scale for Motor and Cognitive Functions (FSMC) was developed and validated in MS patients to fulfill the above-mentioned criteria [14].

In terms of MS disease modifying drugs (DMTs), there are no conclusive data available regarding their efficacy on fatigue symptoms. Studies using first generation DMTs, e.g. interferon (IFN) and glatiramer acetate (GA) have yielded divergent results [15], [16], [17], [18] while a recent publication on the impact of natalizumab on cognition and fatigue [19] showed improvement of both aspects in a two-year follow-up. More data, preferably obtained in a clinical trial setting are warranted, to confirm this observation. The primary objective of the TYNERGY study was to investigate the MS related fatigue during treatment with natalizumab over the course of 12 months after initiation of therapy. The data obtained significantly adds to the knowledge about fatigue during natalizumab treatment of MS, as well as show impact on other aspects of the disease such as quality of life, sleepiness, depression, cognition, and mobility.

## Materials and Methods

The protocol for this trial and supporting STROBE checklist are available as supporting information; see Checklist S1 and Protocol S1.

### Ethics statements

The study was conducted in compliance with Good Clinical Practices (GCP) and the Declaration of Helsinki, and was approved by the institutional ethical review board at the University Hospital of Northern Sweden, Umeå.

### Trial design

The TYNERGY study used a one-armed trial design to evaluate the natalizumab treatment effect on fatigue with a well-defined and validated instrument, the FSMC, designed for use in MS patients. A randomised controlled trial was not performed because at the time of the start of this trial there was no comparator available for the patient population with highly-active MS or with a need of second line MS therapy, which constitutes the patients fulfilling the indication for natalizumab.

### Trial conduct

Consecutive patients prescribed natalizumab at the participating centers gave their written, informed consent to enter the study after the therapy decision was made. Patients were eligible for inclusion in the trial if they were prescribed natalizumab according to national guidelines, aged 18–65 years (both inclusive) at screening and presented with an FSMC sum score of ≥43 (at least mild fatigue at baseline, Table 1). Patients with no symptoms of fatigue, EDSS of ≥6, amphetamine medication or major depression, were not included.

**Table 1:** Cut-off values for the Fatigue Scale for Motor and Cognitive functions (FSMC).

FSMC Sum Score	≥43	Mild fatigue
	≥53	Moderate fatigue
	≥63	Severe fatigue
FSMC Cognitive Score	≥22	Mild cognitive fatigue
	≥28	Moderate cognitive fatigue
	≥34	Severe cognitive fatigue
FSMC Physical Score	≥22	Mild motor fatigue
	≥27	Moderate motor fatigue
	≥32	Severe motor fatigue

The study was performed at 27 centers in Sweden (12), Norway (7), Austria (5) and Denmark (3). The patients attended 5 visits, (at baseline, month 3, 6, 9 and 12) over 12 months. The primary endpoint was fatigue associated with MS as measured by the FSMC total score change at 12 months, compared with baseline. Cut-off values for the clinical categories mild, moderate and severe MS-related fatigue are shown in Table 1. The FSMC allows for the evaluation of the physical component of fatigue, i.e. motor fatigue, as well as the cognitive component, separately, which were also evaluated at every visit and constituted secondary endpoints. To address other important aspects of the function and well-being of MS patients, secondary endpoints were assessed at baseline and at months 6 and 12. They were: Capacity for work (capacity for work questionnaire; CWQ), health related quality of life ((HRQoL) Short Form -12 questionnaire (SF-12)), sleepiness (Epworth Sleepiness Scale; ESS), depression (Center for Epidemiologic Studies Depression scale; CES-D), cognitive impairment (the Paced Auditory Serial Addition Test; PASAT, and Symbol Digit Modalities Test; SDMT), Speed of walking (6 Minute Walk Test; 6MWT), MS disease disability (Expanded Disability Status Scale; EDSS) and amount of walking e.g. a step counter was worn for seven days the week before the study visit.

The DMTs used prior to initialization of natalizumab were documented. All concomitant medications taken during the trial were recorded and special attention was paid to change in symptomatic fatigue therapy, e.g. modafinil and amantadine. Information on relapses, adverse events (AEs) and serious adverse events (SAEs) were collected.

The first patient's first visit was on March 23, 2009 and the last patient's last visit on June 30, 2011. EudraCT number for the Swedish protocol: 2008-008065-35. Clinical Trials.gov identifier: NCT00884481. The study was considered observational in Austria, Norway and Denmark.

### Statistical methods

In order to achieve a power of 90% to detect 25% improvement in fatigue from baseline to month 12 when performing a paired Student's t-test, with the assumption that the standard deviation (SD) was close to 1.0, the sample size was estimated to be 168. The statistical analyses were based on pooled datasets from Sweden, Norway, Denmark and Austria, except SF-12, ESS, CES-D, PASAT and 6MWT, which were not performed in Denmark.

In general, descriptive statistics for continuous variables are presented with Mean, Standard Error (SE), Median, Minimum and Maximum values. Descriptive statistics for discrete variables are presented as percentages. All statistical tests were carried out as two-sided on a 5% level of significance unless otherwise stated.

The primary efficacy analysis was based on an analysis of variance (ANOVA) method on the change from baseline to month 12 for the FSMC total score. Baseline FSMC value was included as a continuous fixed effect.

For the secondary efficacy variables, mixed linear models with repeated measures were done for the FSMC total score, motor score, cognitive score, SF-12, ESS, CES-D, 6MWT and PASAT scores with change from baseline values as response, and baseline value as a continuous fixed effect, and visits as a categorical fixed effect. The covariance matrix was assumed unstructured. Since the EDSS step score was not normally distributed, a non-parametric model and a Hodges-Lehmann confidence interval (CI) with associated p–value from the Wilcoxon-Signed Rank test, were used to assess the change from baseline. The SDMT and Step Counter scores were analysed in a generalised linear mixed model. All statistical analysis and programming were done using SAS v9.2.

## Results

### Patient disposition and datasets analysed

**Figure 1:**
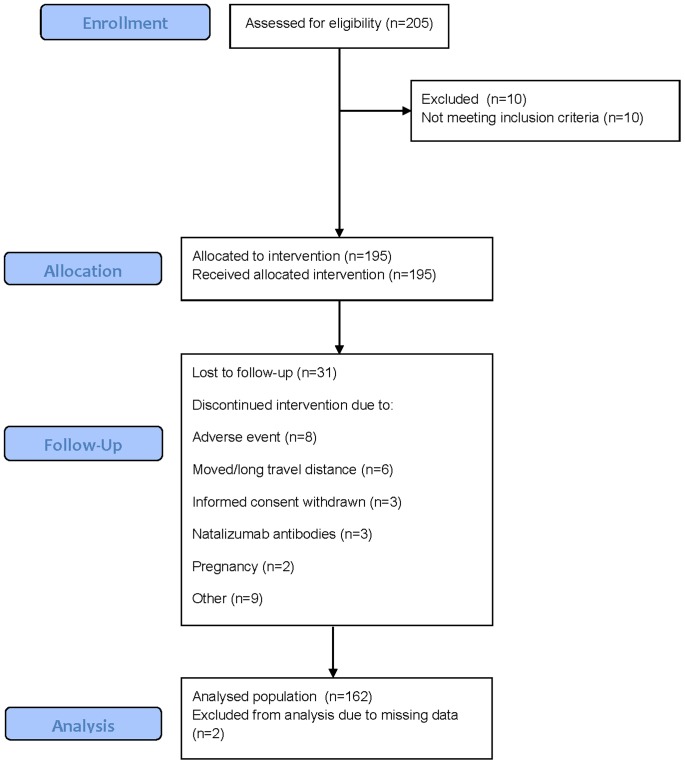
Flow diagram of the TYNERGY trial. The CONSORT flow diagram model showing enrolment, follow-up, and data analysis was applied.

A total of 205 patients were screened and 10 patients were considered screening failures. All patients enrolled constituted the intention to treat population (ITT), (N = 195). A total of 31 withdrawals occurred over the trial, resulting in 164 patients completing the trial. The most common reasons were: adverse event, moved/long travel distance, informed consent withdrawn, antibodies against natalizumab and pregnancy/pregnancy wish (Fig. 1).

### Demographics and baseline characteristics

The study population was representative of what we expected for treatment with natalizumab in clinical practice. More than two-thirds of the 195 patients included were females (71.3%). The average age was 39.7 years, and the average duration of MS was 8.8 years. The average EDSS score at baseline was 3.2 and two-thirds of the patients (132/195) had experienced a relapse 6 months prior to the baseline visit, and 19 within one month before the baseline visit (Table 2).

**Table 2:** Demographics and baseline disease characteristics of the MS patient population included in the TYNERGY study.

Total ITT population N		195
Gender - Female N (%)		139 (71.3)
Race- White N (%)		188 (96.4)
Age (years)	Mean (SD)	39.7 (9.2)
	Median	39.9
	Min, Max	18.3, 63.8
Number with previous DMT treatment (%)		167 (85.6)
IFN beta the month before inclusion (%)		61 (38)
Duration of DMT (years)	Mean (SD)	3.0 (2.7)
	Median	1.9
	Min, Max	0.3, 14.8
Duration of MS (years)	Mean (SD)	8.8 (7.0)
	Median	6.7
	Min, Max	0.2, 30.5
EDSS	Mean (SD)	3.2 (1.2)
	Median	3.0
	Min, Max	0, 7
No of relapses last 6 months		165
No of patients with relapse last 6 months (%)		132 (67.7)
Number of patients with Relapse the month before inclusion (%)		19 (11.7)

SD: Standard deviation.

### Analysis of the primary efficacy endpoint

**Figure 2:**
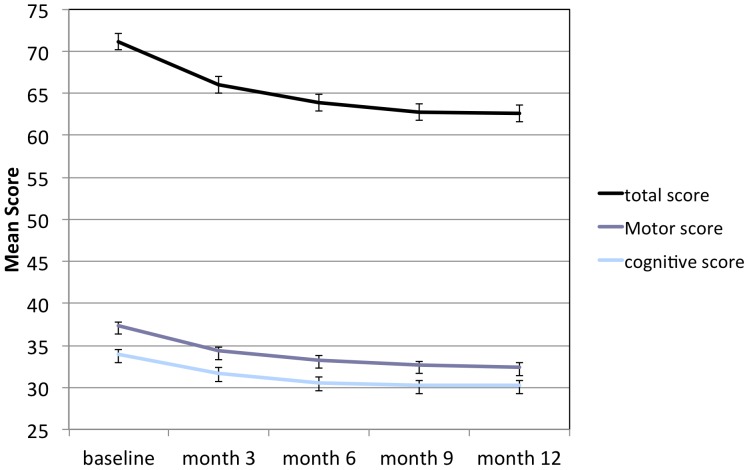
Patient reported fatigue at base line and during the 12 months of natalizumab treatment. A validated questionnaire for MS, the Fatigue Scale for Motor and Cognitive functions (FSMC) allowing separate evaluations of motor and cognitive aspects of fatigue, was used. The total, motor and cognitive FSMC score at base line and month 3, 6, 9 and 12 are shown. Error bars = Standard Error of the Mean.

Fatigue as measured by the change from baseline estimate in FSMC total score showed a significant improvement (p<0.0001) at month 12, with a mean reduction of 9.0 (95% CI −11.2, −6.8) from a baseline mean score of 71.2 (Table 3, Fig. 2). At a population-level this improvement reduced fatigue qualitatively from severe at baseline to moderate at month 12. We regard a mean total score reduction of 9 as clinically meaningful.

**Table 3:** Primary end point results: Fatigue as measured by the FSMC; total, motor and cognitive scores and mean change from baseline to month 12 for the ITT population.

FSMC score at 12 month	N	Mean at Baseline	Mean Change	95% CI	p-value
Total score	162	71.2	−9.00	(−11.2, −6.8)	<0.0001
Motor score	162	37.3	−4.86	(−5.9, −3.8)	<0.0001
Cognitive score	162	33.9	−3.97	(−5.1, −2.9)	<0.0001

FSMC: The Fatigue Scale for Motor and Cognitive functions, CI: Confidence Interval.

### Analyses of secondary efficacy endpoints

The total FSMC score changed in a time dependent manner, with the greatest improvement during the first three months of natalizumab treatment. The mean individual FSMC motor and cognition scores were improved by 4.86 and 3.97 from baseline values of 37.3 and 33.9, respectively (p<0.0001; Table 3, Fig. 1).

The secondary variables HRQoL, SF-12,ESS, CES-D, PASAT, and SDMT, EDSS, and 6MWT all showed significant improvement from baseline to month 12 and the data are shown in Table 4. It is notable that the reduction of the CES-D depression score indicated that the patents improved from an average of 18.3 (equals moderate depression) before start of natalizumab treatment to 14.2 (equals no depression) after 12 months of treatment. The 6 months results of HRQoL, sleepiness, depression, cognition, disability and walking speed were all highly significant (p<0.0008–0.0001) and consistent with the results at 12 months (data not shown). The results from the step-counter test did not show any significant change in the amount of walking from baseline to months 6 or 12. The compliance for the use of the step-counter was poor, leading to a large loss of data and no meaningful conclusions could be drawn from this analysis (data not shown). The CWQ data showed no significant change when measured as overall change in all participating countries although certain data regarding this parameter appeared promising and will be analysed further (data not shown). The majority of the patients (on average 92.6% at each visit) did not receive any fatigue medication throughout the trial, and there were no changes in fatigue-related medication during the trial. The safety profile in the study was comparable to current label for natalizumab.

**Table 4:** Secondary efficacy endpoints displaying significant results; SF-12, ESS, CES-D, PASAT, SDMT, 6MWT and EDSS at baseline and the mean change from baseline to month 12 of the ITT population.

Secondary endpoint at 12 month	N	Mean at Baseline	Mean change	95% CI	P-value
SF-12 Physical (PCS)	137	40.5	3.89	(2.74, 5.03)	<0.0001
SF-12 Mental (MCS)	137	44.8	4.17	(2.72, 5.62)	<0.0001
Sleepiness (ESS)	143	8.8	−1.33	(−1.88, −0.78)	<0.0001
Depression (CES-D)	143	18.3	−3.91	(−5.26, −2.56)	<0.0001
PASAT	130	40.0	4.84	(3.49, 6.19)	<0.0001
SDMT (% change)	143	48.0	9.1%	(6, 12)	<0.0001
6MWT (meters)	135	452	20.6	(7.9, 33.3)	0.0016
EDSS	163	3.2	−0.75	(−0.75, −0.5)	<0.0001

ITT: Intention to treat, CI: Confidence Interval.

### Sensitivity analyses

A number of sensitivity analyses were performed to check for possible bias of the results. These analyses showed that age, gender, EDSS at baseline, relapse within one month before initiation of natalizumab (Y/N), number of relapses within 6 months prior initiation of natalizumab, time from the last relapse to the first natalizumab infusion, and previous MS therapy (e.g. IFN beta) within one month were not associated with primary endpoint of this study. Multivariate regression model showed that patients having higher FSMC total score at baseline were more likely to improve after 12 months of natalizumab treatment (Odds Ratio (OR) = 1.084, 95% CI [1.01, 1.16], p = 0.02), and patients having lower depression score at baseline were more likely to improve after 12 months of treatment (OR = 0.905, 95% CI [0.84, 0.98], p = 0.01). However, sensitivity analysis adjusted for fatigue and depression baseline scores showed consistent results as previous findings, with a mean reduction of 9.5 (95% CI −11.9, −7.1). Last observation carried forward (LOCF) analyzing the FSMC showed consistent results (data not shown).

## Discussion

This one-armed, clinical trial showed that there was an improvement of fatigue after 12 months of natalizumab treatment. Both the physical and the mental component of fatigue improved in a time-dependent manner. The average improvement corresponded to a reduction from severe to moderate fatigue, according to the FSMC scale [14], which is presently recommended as the best scale for multidimensional analysis of MS fatigue [20]. Likewise, several of the secondary outcome variables measuring depression, cognitive function, sleepiness, and general quality of life, displayed statistically significant improvements over the 12-month study period.

No controlled studies have been reported that have primarily addressed impact of DMTs on fatigue, and currently there is no consensus regarding how therapies directed against the neuroinflammatory component affect MS-related fatigue. Some uncontrolled studies indicate, however, that the use of DMTs may positively influence fatigue.

In two studies of glatiramer acetate (GA), the fatigue impact scale (FIS) [16] and the modified FIS (MFIS) [18] were utilised. While the results of these studies were positive, the improvements observed were modest. In the study by Metz et al., the proportion of patients improving by more than one SD was 25% in the GA-treated group, which was significantly different than the IFNβ-treated group (14%. In a study by Ziemsen et al. 291 treatment-naïve patients were evaluated with MFIS at baseline and after one year of GA treatment. The MFIS decreased from 34.6 to 27.0. However, since the cut-off value for fatigue with the MFIS scale is 38, the clinical meaningfulness of this reduction is hard to interpret. There is no evidence to indicate whether treatment with IFNs have an effect on fatigue. In one study assessing fatigue while initiating IFN treatment in MS patients, there was an initial tendency for improvement of fatigue measured by MFIS in the first 6 months, but reverted to baseline levels in two of the three IFN groups studied [15]. There are no studies where fatigue has been specifically investigated during fingolimod treatment.

Two published studies have longitudinally studied the effect of natalizumab on MS fatigue as the primary outcome variable, and they both reported a favorable outcome [19], [21]. In the study by Putzki et al. 42 patients were followed over 6 months, MFIS and fatigue severity scale (FSS) improved significantly. In the recently published study by Iaffaldano et al., 100 patients were followed for one year and 53 patients were followed for two years, the mean FSS score at baseline was 4.01 and 45% of the patients scored >4.6, the cut-off level for fatigue interfering with daily activities [19]. After 12 months of natalizumab treatment, these scores were significantly reduced to 3.61 and 29%, respectively. Finally, a cross-sectional study comparing the level of fatigue between 49 patients receiving natalizumab with 53 patients treated with IFN/GA matched for age, gender and disability indicated a lower level of fatigue in the natalizumab-treated group base on all MFIS subscales except the psychosocial domain [22]. Prevalence of severe fatigue was 34.7% in the natalizumab group compared with 51% for IFN/GA group.

The TYNERGY trial is the most comprehensive and systematic study to evaluate the effects of highly-active immunomodulatory treatment on fatigue, as well as a large set of related symptoms crucial for the well-being of MS patients. The study was performed in a clinical trial setting with all statistical analyses predefined and thus eliminating spurious post-hoc observations of questionable importance. A broad repertoire of these predefined scales measuring HRQoL, sleepiness, cognitive functions, walking speed and depression displayed robust and significant improvement during 12 months treatment with natalizumab.

It was not considered ethical to include a control arm, as these patients had high disease activity, in addition to no other second-line treatment available at the start of the study. It is therefore a risk that our data, at least partly, can be explained by the strong placebo effect from starting a new and more efficient treatment and hence, one must be cautious with the final interpretation of the results.

Another confounder could be that the patients experienced a temporary increased level of fatigue due to relapse at the time of natalizumab treatment start. We therefore analysed this factor and could determine that the time from last relapse to treatment start did not influence the positive response on fatigue observed after the start of natalizumab treatment. Another possible source of error could be that the improvement of fatigue simply was a relief of side effects from IFN treatment, the AE of which may result in increased fatigue. Also in this aspect, correction for type of previous DMT treatment, including no treatment at all, did not diminish the positive effect of natalizumab treatment on fatigue. It is therefore reasonable to conclude that the main reason to the suspected improvement in MS-related fatigue observed in this study was possibly a direct result of the improved inflammatory control imposed by the initiation of natalizumab treatment.

It has been suggested that MS fatigue is closely related to immunological factors. During relapse patients often report increased fatigue symptoms and some immunomodulators are known to produce fatigue as a side effect [23]. In MS, peripheral cytokines are able to enter the brain since the blood-brain barrier in certain regions of the brain is either less restricted or absent [24]. In addition, cytokines themselves have the potential to contribute to the destruction of the blood brain barrier and hereby facilitate entrance of inflammatory cells [25]. Certain cytokines are discussed to be related to fatigue. Interleukin (IL) -1α, IL-1β, IL-6, tumour necrosis factor (TNF) α as well as IFNγ have been shown to be related to fatigue-like symptoms in animals [26]. In MS patients, TNFα and IFNγ have been shown to be the variables most likely to be associated with fatigue [27], [28], [29]. The fact that natalizumab seemed to reduce fatigue symptoms in our cohort of patients might be related to the interference with inflammatory cells relevant for the genesis of fatigue in MS.

Natalizumab has a well-documented effect on direct disease-related parameters such as relapse rate, disease progression and inflammatory activity as measured by magnetic resonance imaging (MRI) [30], [31]. It is a common experience among clinicians working with MS patients that the consequences of the disease affecting quality of life are of great importance and should therefore be taken into account in the evaluation of different DMTs. We therefore believe that the results of the TYNERGY trial add important information to the pivotal studies regarding these modalities of treatment efficacy.

In conclusion, this study indicates that initiation of natalizumab in MS improves relevant factors related to quality of life and well-being. The one-armed study design was a prerequisite for conducting a study in second line MS, as no alternative treatment has been available for highly active MS or patients with breakthrough disease activity, until recently. The results of the TYNERGY study, together with the results from the other cohort studies previously discussed, suggests that natalizumab may improve fatigue in MS patients. These results should be regarded as preliminary, and a comparative trial to verify the findings is warranted.

## Supporting Information

Protocol S1 **Trial protocol.**
(PDF)

Checklist S1 **STROBE checklist.**
(DOC)
