# Supplementary material for: Natalizumab Treatment Reduces Fatigue in Multiple Sclerosis. Results from the TYNERGY Trial; A Study in the Real Life Setting
Source: PLoS One. 2013 Mar 21;8(3):e58643. doi: 10.1371/journal.pone.0058643 (PMC3605436; doi:10.1371/journal.pone.0058643)
Supplement: Protocol S1 — Trial protocol. (PDF) [file pone.0058643.s001.pdf]

Clinical Trial Protocol

TYNERGY

A multi-centre and prospective trial to evaluate the effects on multiple sclerosis related fatigue during treatment with Tysabri® in patients with relapsing remitting multiple sclerosis over the course of 12 months

Trial ID:	TYNERGY
EudraCT No:	2008-008065-35
Trial phase:	Phase IV, post-marketing trial
Sponsor name:	Biogen Idec A/S<br>Ørestads Boulevard 67<br>DK-2300 Copenhagen S, Denmark
Date of Protocol:	2 December 2008

Confidentiality Notice

This document contains confidential information of Biogen Idec A/S.
This document must not be disclosed to anyone other than the trial staff and members of the independent ethics committee/institutional review board or regulatory authorities.
The information in this document cannot be used for any purpose other than the evaluation or conduct of the trial investigation without the prior written consent of Biogen Idec A/S.

APPROVAL OF PROTOCOL

Sponsor:	Janne Harder, MD, Medical Director<br>Biogen Idec A/S<br>Ørestads Boulevard 67, DK-2300 Copenhagen S<br>Denmark<br>Phone no.: +45 7741 5794

Name (print)	Signature	Date

Coordinating investigator	Anders Svenningsson, MD<br>Umeå University Hospital<br>Neurocentrum<br>SE-901 85 Umeå,<br>Sweden<br>Phone no.: +46 (0)90 78 50 00

Name (print)	Signature	Date

Author of the Protocol:	Pia Berntsson, PhD<br>aCROnordic A/S<br>Kogle Allé 5, DK-2970 Hørsholm<br>Denmark<br>Phone no.: +45 45 16 88 00

Name (print)	Signature	Date

Responsible Statistician:	Christian Max Møller, PhD<br>aCROnordic A/S<br>Kogle Allé 5, DK-2970 Hørsholm<br>Denmark<br>Phone no.: +45 45 16 88 00

Name (print)	Signature	Date

Chief Medical Officer:	Mads Engelmann , MD, PhD<br>aCROnordic A/S<br>Kogle Allé 5, DK-2970 Hørsholm<br>Denmark<br>Phone no.: +45 45 16 88 00

Name (print)	Signature	Date

AGREEMENT ON THE PROTOCOL

The investigator agrees to conduct the trial as outlined in this protocol with reference to national/local regulations and in accordance with current Good Clinical Practice (GCP) guidelines (1). Any modification to the protocol must be approved in writing by the investigator, Biogen Idec A/S, Regulatory Authorities and the Independent Ethics Committee (IEC) as required by national regulations.

The investigator agrees, by written consent to this protocol, to fully co-operate with monitoring and audit checks by allowing direct access to patients' personal data and records, including source data, by authorised individuals representing Biogen Idec A/S or Regulatory Authorities.

Approved consent in writing:

Signature: ______________________________ Date: ____________________

Investigator

Name: ______________________________

(Print)

Table of Contents

Page

1 Abbreviation and Definition of Terms **7**
1.1 Abbreviations 7
1.2 Definitions of Terms 8
Protocol Summary **10**
Rationale **10**
Objective **10**
Endpoints **10**
Trial Design **10**
Trial Population **10**
Trial Assessments **10**
2 Introduction **11**
2.1 Background 11
2.2 Rationale 12
2.3 GCP Compliance Statement 12
3 Objectives **12**
3.1 Primary Objective 12
3.2 Secondary Objective 12
4 Endpoints **13**
4.1 Primary Endpoint 13
4.2 Secondary Endpoints 13
5 Trial Design **14**
5.1 Description of Trial Design 14
5.2 Justification of Trial Design 14
6 Trial Visits **14**
7 Trial Population **16**
7.1 Number of Subjects 16
7.2 Eligibility Criteria 16
7.2.1 Inclusion Criteria 16
7.2.2 Exclusion Criteria 16
7.2.3 Other Eligibility Criteria Considerations 16
7.2.4 Screening Failures 17
7.2.5 Subject Completion 17
7.2.6 Withdrawal Criteria and Early Termination 17
8 Trial Assessments **17**
8.1 Demographic and Baseline Assessments 17
8.2 Efficacy 18
8.3 Safety 18
9 Tysabri® **18**

10 Concomitant Medications and Non-Drug Therapies ... 18
10.1 Prohibited Medications ... 19
11 Adverse Event and Serious Adverse Event Collection, Recording and Reporting ... 19
11.1 Definition of Adverse Events and Serious Adverse Events ... 19
11.1.1 Definition of an Adverse Event ... 19
11.1.2 Definition of a Serious Adverse Event ... 19
11.1.3 Disease-Related Events and/or Disease-Related Outcomes Not Qualifying as SAEs ... 20
11.1.4 Clinical Laboratory Abnormalities and Other Abnormal Assessments as AEs and SAEs ... 20
11.2 Reporting of Adverse Events ... 20
11.3 Time Period, and Frequency of Detecting AEs and SAEs ... 22
11.4 Pregnancies and Breast Feeding ... 22
12 Data Analysis and Statistical Considerations ... 23
12.1 Hypotheses ... 23
12.2 Sample Size Considerations ... 23
12.3 Data Analysis Considerations ... 23
12.3.1 Analysis Data Sets and Populations ... 23
12.3.2 Treatment Comparisons ... 23
12.3.2.1 Primary Comparisons of Interest ... 23
12.3.2.2 Other Comparisons of Interest ... 23
12.3.3 Interim Analysis ... 24
12.3.4 Key Elements of Analysis Plan ... 24
12.3.4.1 Efficacy Analyses ... 24
12.3.4.2 Safety Analyses ... 25
13 Trial Administration ... 25
13.1 Regulatory and Ethical Considerations, Including the Informed Consent Process ... 25
13.2 Definition of Source Data ... 26
13.3 Direct Access ... 26
13.4 Quality Control (Trial Monitoring) ... 26
13.5 Quality Assurance ... 27
13.6 Trial and Site Closure ... 27
13.7 Records Retention ... 27
13.8 Provision of Trial Results and Information to Investigators ... 28
13.9 Data Management ... 28
13.10 End of Trial Definition ... 28
13.11 Finance and Insurance ... 28
13.12 Publication Policy ... 29
14 References ... 30

List of Appendices

Appendix 1: **Trial flow chart**

Appendix 2: **FSMC**

Appendix 3: **CWQ**

Appendix 4: **SF-12**

Appendix 5: **ESS**

Appendix 6: **CES-D**

Appendix 7: **PASAT**

Appendix 8: **SDMT (6MWT)**

Appendix 9: **BS-CR10 (6MWT)**

Appendix 10: **EDSS**

Appendix 11: **Step counter**

1 Abbreviation and Definition of Terms

1.1 Abbreviations

6MWT	6 Minutes Walk Test
AE	Adverse event
BS-CR10	Borg Scale CR10
CES-D	Center for Epidemiologic Studies Depression Scale
CI	Confidence Interval
CNS	Central Nervous System
CRF	Case Report Form
CRO	Contract Research Organisation
CWQ	Capacity for Work Questionnaire
DMT	Disease Modifying Therapy
EEA	European Economic Area
EDSS	Expanded Disability Status Scale
ESS	Epworth Sleepiness Scale
FSMC	Fatigue Scale for Motor and Cognitive functions
GCP	Good Clinical Practice
HRQoL	Health Related Quality of Life
ICF	Informed Consent Form
IEC	Independent Ethics Committee
IFN-β	Interferon-β
ITT	Intention to Treat
MedDRA	Medical Dictionary for Regulatory Activities
MS	Multiple Sclerosis
NA	Not Applicable

ND	Not Done
PASAT	The Paced Auditory Serial Addition Test
PRN	Pro Re Nata
QoL	Quality of Life
RRMS	Relapsing-Remitting Multiple Sclerosis
SAE	Serious Adverse Event
SAP	Statistical Analysis Plan
SAR	Serious Adverse Reaction
SD	Standard Deviation
SDMT	Symbol Digit Modalities Test
SF-12	Short Form -12 questions
SmPC	Summary of Product Characteristics
SUSAR	Suspected Unexpected Serious Adverse
VCAM-1	Vascular Cell Adhesion Molecule-1

1.2 Definitions of Terms

Baseline	The ‘baseline’ value is the value measured at baseline visit (month 0) before first administration of Tysabri®.
End-of-trial	The completion of the last visit of the last trial subject in any participating centre.
Pro Re Nata (PRN)	When necessary; as the occasion arises.
Relapse	The development of new or the exacerbation of existing neuro-logical symptoms or sign, in the absence of fever, persisting for more than 48 hours and with a previous period for more than 30 days with a stable or an improving condition.
Screening	Time period starting at signing of ICF and until inclusion at baseline visit (month 0).
Step counter	A device for measuring the amount of steps a person has taken when actively walking.

SUSAR	All AEs that are suspected to be related to an investigational medicinal product and that are both unexpected and serious are considered SUSARs.
Tysabri®	Natalizumab

Protocol Summary

Rationale

Different studies have shown that fatigue is a problem in 75-95 % of multiple sclerosis (MS) patients and many MS patients report fatigue as one of their most disabling symptoms which contributes to cognitive and physical difficulties. Treatment that can decrease fatigue will improve the quality of life (QoL) for this group of patients. Small studies and anecdotal reports have suggested that Tysabri®, as opposed to other disease modifying therapies (DMTs), can decrease fatigue in MS patients. Data from a more substantial trial population and a well-defined fatigue scale is warranted to verify these observations and to investigate the MS related fatigue during treatment with Tysabri®.

Objective

The primary objective is to investigate the MS related fatigue during Tysabri® treatment in MS patients over the course of 12 months.

Endpoints

The endpoints in the trial are fatigue, capacity for work, health related quality of life (HRQoL), sleepiness, depression, cognitive impairment, physical activity induced exhaustion, speed of walking, status of MS disease progression and amount of walking.

Trial Design

The trial is a multi-centre and prospective 12 month trial which aims to investigate the MS related fatigue during treatment with Tysabri® in subjects with relapsing remitting MS (RRMS) over the course of 12 months.

Trial Population

Approximately 200 subjects will be recruited for the trial from 29 centres in Austria (5 centres), Denmark (4 centres), Finland (5 centres), Norway (5 centres) and Sweden (10 centres) with approximately 7 subjects from each centre.

Trial Assessments

The trial period for the subjects is 12 months and they will attend 5 visits during this time period. No trial drug is used in the trial and only subjects already prescribed Tysabri® and who have not initiated treatment yet are considered eligible for the trial. Fatigue and other related endpoints are measured using different questionnaires and tests in the trial.

2 Introduction

2.1 Background

Multiple sclerosis (MS) related fatigue is considered as the most common symptom of MS impacting 75-95 % of the patients and 50-60 % of the MS patients report fatigue as their major problem (2-5). Fatigue, occurring in the apparent absence of physical symptoms may handicap patients with MS, who otherwise have no physical impairment or disability but nevertheless severely limiting their ability to participate in every day activities. The impact of fatigue on daily life is substantial, as it prevents sustained physical and mental exertion, limits work and social role performance and is related to less satisfaction with quality of life (QoL). Beside exercise and energy saving strategies, several pharmacological drugs have been evaluated for their ability to reduce MS-related fatigue. Currently, the dopaminergic drug amandantine, amphetamine-related pemoline and wake-promoting modafinil are used to decrease MS-related fatigue.

Patients with MS can also experience some degree of fatigue from time to time which may be aggravated by heat and physical efforts and may worsen throughout the day. Furthermore, fatigue may also be influenced by relapses, medication, depression and sleeping disorders (6).

Assessment of fatigue is difficult as it is not only based on experienced subjectivity but also composed of a motor and cognitive aspect. More than a dozen fatigue questionnaires have been developed and most of them do not differentiate sufficiently between motor and cognitive fatigue and thereby showing methodological limitations. A fatigue scale for motor and cognitive functions (FSMC) has been developed and validated by Penner et al. (7). FSMC differentiates between motor and cognitive fatigue and is less susceptible to confounding by depression. Thus, FSMC is more specific in measuring MS-related fatigue.

Natalizumab is a new monoclonal antibody for treatment of relapsing-remitting multiple sclerosis (RRMS) which is marketed as Tysabri® by Biogen Idec A/S. It binds to the $\alpha4$ unit on the surface of the leukocytes and inhibits the leukocytes from binding to the Vascular Cell Adhesion Molecule-1 (VCAM-1) receptor on the surface of the vascular endothelial cells. Leukocytes are thereby inhibited to migrate over the blood brain barrier, thus preventing the inflammation in the central nervous system (CNS). Tysabri® has been documented to significantly reduce the relapse rate and disability progression in MS (8;9). Tysabri® has in the phase III trial showed improved "well-being" including fatigue by 70 %. In the Sentinel trial, natalizumab was combined with interferon-β (IFN-β) and improvement in fatigue was demonstrated using a modified fatigue impact scale (MFIS). At ECTRIMS 2007, Putzki et al. presented data from 34 patients showing that natalizumab improved MS related fatigue by using the MFIS, visual analogue scale (VAS) and fatigue severity scale (FSS) (10).

MS patients experiencing fatigue are most often either working reduced time, might be full time sick-listed or even unemployed. From a health economic perspective, fatigue is one of the factors that contribute to the economic burden of MS and a treatment that might improve the capacity for work will provide a strong social rationale for prioritizing resources within the disease modifying therapy (DMT). It has been reported that Tysabri® has decreased fatigue in MS patients; however, a structured measurement is needed in order to document this observation. It would be of great importance if Tysabri® not

only could prevent disease progression but also relieve fatigue in MS patient, thus improving the quality of life for individuals.

2.2 Rationale

The trial aims to investigate the MS related fatigue during Tysabri® treatment by using a scale which differentiates between motor and cognitive fatigue.

Different studies have shown that fatigue is a problem in 75-95 % of MS patients and many of those patients report fatigue as one of their most disabling symptoms which contributes to cognitive and physical difficulties. Treatment that decreases fatigue will improve the QoL for this group of patients. Small studies and anecdotal reports have suggested that Tysabri®, as opposed to other DMTs, can decrease fatigue in MS patients. Data from a more substantial trial population is warranted to verify these observations and to investigate the MS related fatigue during treatment with Tysabri®.

2.3 GCP Compliance Statement

The trial will be conducted in accordance with current Good Clinical Practice (GCP) guidelines. The clinical trial protocol and any future protocol amendments will comply with the ICH Harmonised Tripartite Guideline for Good Clinical Practice (1) in general and section 6 in particular as well as the ICH E9 guideline on Statistical Principles for Clinical Trials (11).

3 Objectives

3.1 Primary Objective

The primary objective of this trial is to investigate the MS related fatigue during treatment with Tysabri® as measured by changes in the fatigue scale for motor and cognitive functions (FMSC) over the course of 12 months.

3.2 Secondary Objective

The secondary objectives are:

- to investigate changes in fatigue, capacity for work, HRQoL, sleepiness, cognitive impairment, physically activity induced exhaustion, speed of walking, status of MS disease progression and amount of walking at different time points after initiation of Tysabri® treatment in subjects diagnosed with RRMS. Changes in fatigue are measured at 3, 6 and 9 months, whereas changes in capacity for work, HRQoL, sleepiness, cognitive impairment, physical activity induced exhaustion, speed of walking, status of MS disease progression and amount of walking are measured at 6 and 12 months.
- to investigate correlation between fatigue and cognitive impairment, depression and physically activity induced exhaustion and status of MS disease progression in subjects at baseline, 6 and 12 month of treatment with Tysabri®.

- to document any changes in fatigue related medication.

4 Endpoints

The endpoints in the trial are fatigue associated with MS, capacity for work, HRQoL, sleepiness, depression, cognitive impairment, physical activity induced exhaustion, speed of walking, status of MS disease progression and amount of walking. Each visit should take place at the same time of day to avoid influence on fatigue depending on circadian rhythm.

4.1 Primary Endpoint

The primary endpoint is fatigue associated with MS (FSMC) measured at 0 and 12 months after initiating treatment with Tysabri®.

4.2 Secondary Endpoints

The secondary endpoint measured 3, 6 and 9 months after initiating treatment with Tysabri® is:

- Fatigue associated with MS (FSMC)

The secondary endpoints measured 0, 6 and 12 months after initiating treatment with Tysabri® are:

- Capacity for work (capacity for work questionnaire (CWQ))
- HRQoL (short form -12 questions (SF-12))
- Sleepiness (Epworth sleepiness scale (ESS))
- Depression (center for epidemiologic studies depression scale (CES-D)
- Cognitive impairment (the paced auditory serial addition test (PASAT) and symbol digit modalities test (SDMT). SDMT is done twice at each visit, before and after the 6 minute walk test (6MWT)
- Physical activity induced exhaustion (Borg scale CR10 (BS-CR10)). The BS-CR10 is graded twice at each visit, before and after the 6MWT
- Speed of walking (6MWT)
- Status of MS disease progression (expanded disability status scale (EDSS))
- Amount of walking (step counter)

Any changes in fatigue related medication are also documented together with DMTs used Information on relapses, adverse events (AEs) and serious adverse events (SAEs) are collected for assessing their effects on fatigue.

5 Trial Design

5.1 Description of Trial Design

This is a multi-centre and prospective trial which aims to investigate the MS related fatigue at the time of the administration of the first dose of Tysabri® (at screening/baseline) and end 12 months after this time-point in subjects with RRMS. A trial flow chart is included in Appendix 1.

200 subjects are planned to be screened over a recruitment period of 12 months. The subjects should attend 5 visits during a time period of 12 months. The first visit of the first subject is planned to be in the first quarter of 2009, and the last visit of the last subject in the first quarter of 2011.

5.2 Justification of Trial Design

In order to investigate MS related fatigue in subjects treated with Tysabri® according to clinical practice an trial design where Tysabri® is prescribed before the subjects enter the trial was chosen. The decision to prescribe Tysabri® to patients is clearly separated from any trial activities and from the decision of including subjects in the trial. The test assessments start at the time of the administration of the first dose of Tysabri® (at screening/baseline) and end 12 months after this time-point.

6 Trial Visits

The subjects should attend 5 visits during a maximal time period of 12 months. At start of infusion (month 0) baseline measurements are performed and during the following 4 visits (3, 6, 9 and 12 months) changes from baseline are measured (Panel 1).

Panel 1. Tests and endpoints during the trial with references.

Test	Endpoint	Month	Visit	Reference
FSMC	Fatigue	 0<br>3<br>6<br>9<br>12	Screening/<br>Baseline<br>1<br>2<br>3<br>4	(Appendix 2)<br>(6;7)
CWQ	Capacity for work	 0<br>6<br>12	Screening/<br>Baseline<br>2<br>4	(Appendix 3)
SF-12	HRQoL	 0<br>6<br>12	Screening/<br>Baseline<br>2<br>4	(Appendix 4)<br>(12)
ESS	Sleepiness	 0<br>6<br>12	Screening/<br>Baseline<br>2<br>4	(Appendix 5)<br>(5)
CES-D	Depression	 0<br>6<br>12	Screening/<br>Baseline<br>2<br>4	(Appendix 6)<br>(13)
PASAT	Cognitive impairment	 0<br>6<br>12	Screening/<br>Baseline<br>2<br>4	(Appendix 7)<br>(14;15)
SDMT (6MWT)[1]	Cognitive impairment	 0<br>6<br>12	Screening/<br>Baseline<br>2<br>4	(Appendix 8)<br>(16)
BS-CR10 (6MWT) [1]	Physical activity induced exhaustion	 0<br>6<br>12	Screening/<br>Baseline<br>2<br>4	(Appendix 9)<br>(17-19)
6MWT	Speed of walking	 0<br>6<br>12	Screening/<br>Baseline<br>2<br>4	Appendices 8, 9 (17-19)
EDSS	Status of MS disease progression	 0<br>6<br>12	Screening/<br>Baseline<br>2<br>4	(Appendix 10)<br>(20;21)
Step counter	Amount of walking	 0<br>6<br>12	Screening/<br>Baseline<br>2<br>4	(Appendix 11)<br>(22)

1) SDMT and BS-CR10 are performed twice at each visit, before and after the 6MWT. The 6MWT is only to be performed once at each visit.

7 Trial Population

The trial population will consist of subjects with RRMS diagnosed using 2005 revision to the McDonald criteria (23) and who have been prescribed Tysabri® according to local guidelines, but not started treatment yet.

7.1 Number of Subjects

200 subjects are planned to be screened in the trial from 29 centres in Austria (5 centres), Denmark (4 centres), Finland (5 centres), Norway (5 centres) and Sweden (10 centres) with approximately 7 subjects from each centre. The total number to be enrolled in the study is 168 subjects.

7.2 Eligibility Criteria

7.2.1 Inclusion Criteria

A subject will be eligible for inclusion in this trial if all of the following criteria apply:

- Age $\geq$ 18 years and age $\leq$ 65 years at screening
- Subjects who have been prescribed Tysabri® according to national guidelines but not yet started treatment
- Signed informed consent form (ICF)
- FSMC sum score $\geq$ 43 at baseline (mild fatigue)

7.2.2 Exclusion Criteria

A subject will not be eligible for inclusion in this trial if any of the following criteria applies:

- FSMC sum score < 43 at baseline
- History of treatment with Tysabri®
- EDSS ≥ 6 at baseline
- Amphetamine as medication
- Major depression

7.2.3 Other Eligibility Criteria Considerations

To assess any potential impact on subject eligibility with regard to safety, the investigator must refer to the following document for detailed information regarding warnings, precautions, contraindications, adverse events (AEs) and other significant data pertaining to Tysabri® : Approved national summary of product characteristics (SmPC).

7.2.4 Screening Failures

In case of screening failure, the investigator will fill in the 'End-of-Trial Form' (incl. screening failure) in the Case Report Form (CRF), stating reason for subject not fulfilling criteria.

7.2.5 Subject Completion

On completion of the trial, the investigator will fill in the 'End-of-Trial Form' in the CRF.

7.2.6 Withdrawal Criteria and Early Termination

The subjects have the right to withdraw from the trial at any time and for any reason without prejudice to his or her future medical care by the physician or hospital. The investigator and Biogen Idec A/S also have the right to withdraw subjects from the trial in the event of change in eligibility or other reasons.

In case of withdrawal, the investigator will fill in the 'End-of-Trial Form' in the CRF, stating the reason for termination.

Discontinuation criteria for individual subjects:

- Withdrawal of consent
- Stopped treatment of Tysabri®

Withdrawn subjects are not to be replaced. Information on current medication, SAEs, AEs and relapses are to be collected from withdrawn subjects.

Biogen Idec A/S may terminate the trial at any time.

8 Trial Assessments

8.1 Demographic and Baseline Assessments

Demographic information such as gender, age and ethnic origin will be collected in addition to baseline values for the efficacy and safety assessments described in section 8.2-8.3.

Other baseline assessments are:

- Signed and dated ICF
- Inclusion/exclusion criteria
- Disease history/duration
- Current medication
- Information about relapses
- History of DMT (e.g. INF-β and glatiramer acetate)

8.2 Efficacy

The following efficacy assessments will be performed at 3, 6, 9 and 12 months after initiating treatment with Tysabri® compared to baseline:

- Changes in fatigue (FSMC)
- Changes in any fatigue related medication

Changes in following endpoints at 6 and 12 months after initiating treatment with Tysabri® compared to baseline:

- Capacity for work (CWQ)
- HRQoL (SF-12)
- Sleepiness (ESS)
- Depression (CES-D)
- Cognitive impairment (PASAT and SDMT)
- Physical activity induced exhaustion (BS-CR10)
- Speed of walking (6MWT)
- Status of MS disease progression (EDSS)
- Amount of walking (Step counter)

8.3 Safety

AEs will be collected and evaluated for relation to current medication, trial procedures, seriousness and expectedness. They will be reported to authorities and followed-up according to local requirements as described in section 11.

Information about relapses will also be collected throughout the trial.

9 Tysabri®

Subjects enrolled in the trial should follow the Tysabri® treatment indication and guideline stated in the SmPC and the treatment determined by their physician. The decision to prescribe Tysabri® to patients is clearly separated from any trial related activities and from the decision of including subjects in the trial. See national SmPC for more information about Tysabri®.

10 Concomitant Medications and Non-Drug Therapies

All concomitant medications taken during the trial will be recorded in the CRF. The minimum requirement is that the drug name and dates of administration are recorded. The subjects should follow the treatment determined by their physician. Special attention should be paid to symptomatic fatigue therapy (e.g. modafinil and amandantine) and any adjustment in dose during the trial should be recorded in the CRF. Fatigue treatment taken on a regular basis is continued, in case of fatigue medication taken as p.r.n then it is stopped 48 hours before measurements.

10.1 Prohibited Medications

Medication containing amphetamine is not allowed during the trial.

11 Adverse Event and Serious Adverse Event Collection, Recording and Reporting

The investigator is responsible for detection and documentation of events meeting the criteria and definition of an adverse event (AE) or serious adverse event (SAE), as provided in this protocol. During the trial when there is a safety evaluation, the investigator or site staff will be responsible for reporting AEs and SAEs, as detailed in this section of the protocol. Clinically significant AEs considered by the investigator or the sponsor to be related to treatment will be followed until resolved or considered stable.

It will be left to the investigator's clinical judgment to determine whether an AE is related and of sufficient severity to require the subject's removal from treatment or from the trial. A subject may also voluntarily withdraw from treatment due to what he or she perceives as an intolerable AE. If either of these situations arises, the subject should be strongly encouraged to undergo an end of trial assessment and be under medical supervision until symptoms cease or the condition becomes stable.

11.1 Definition of Adverse Events and Serious Adverse Events

11.1.1 Definition of an Adverse Event

An AE is defined as any untoward medical occurrence in a clinical trial subject administered a medicinal product and which does not necessarily have a causal relationship with this treatment.

An AE can therefore be any unfavourable and unintended sign (including an abnormal laboratory finding), symptom, or disease (new or exacerbated) temporally associated with the use of a medicinal product, whether or not considered related to the product. For marketed medicinal products, this also includes failure to produce expected benefits (i.e. lack of efficacy), abuse or misuse.

11.1.2 Definition of a Serious Adverse Event

A SAE is defined as any untoward medical occurrence or effect, that at any dose:

- Results in death
- Is life threatening
- Requires hospitalization or prolongation of existing hospitalisation
- Results in persistent or significant disability or incapacity
- Is a congenital anomaly or a birth defect
- Is an other significant medical hazard

An AE necessitating hospitalisation meets the regulatory definition for "serious" if the inpatient hospital admission includes a minimum of an overnight stay in a health care facility. Any AE that does not meet one of the definitions of serious (e.g. an AE requiring an

emergency room visit, outpatient surgery or requires urgent investigation) may be considered by the investigator to meet the "other significant medical hazard" criterion for classification as a SAE. Examples include allergic bronchospasm, convulsions and blood dyscrasias.

11.1.3 Disease-Related Events and/or Disease-Related Outcomes Not Qualifying as SAEs

Relapses or symptoms associated to MS is not considered as an AE and should not be recorded as such irrespective of hospitalisation. Planned operations or examinations requiring hospitalisations are not considered as SAE, only complications associated with the procedures are considered as AEs or SAEs.

11.1.4 Clinical Laboratory Abnormalities and Other Abnormal Assessments as AEs and SAEs

Abnormal laboratory findings (e.g. clinical chemistry, haematology and urinalysis) or other abnormal assessments that are judged by the investigator as clinically significant will be recorded as AEs or SAEs if they meet the definition of an AE or SAE. Clinically significant abnormal laboratory findings or other abnormal assessments that are detected during the trial or are present at baseline and significantly worsen following the start of the trial will be reported as AEs or SAEs. However, clinically significant abnormal laboratory findings or other abnormal assessments that are associated with the disease being studied, unless judged by the investigator as more severe than expected for the subject's condition, or that are present or detected at the start of the trial and do not worsen, will not be reported as AEs or SAEs.

The investigator will exercise his or her medical and scientific judgment in deciding whether an abnormal laboratory finding or other abnormal assessment is clinically significant.

11.2 Reporting of Adverse Events

The investigator is responsible for ensuring that all AEs (as defined in Section 11.1) observed by the investigator or reported by subjects are properly captured in the subjects' medical record. In addition, the investigator is responsible for ensuring that all AEs captured on the subjects' medical records are reported on the CRF. If a subject is permanently withdrawn from the trial because of a SAE, this information must be included in the initial or follow-up SAE report form, as well as the End-of-Trial form. In case of withdrawal due to an AE, then the AE report form and as well as the End of Trial form will be completed.

AEs either reported by the patient or observed by the investigator should be described in the following manner:

- The nature of the event will be described in precise, standard medical terminology (i.e. not necessarily the exact words used by the patient). If known, a specific diagnosis should be stated.

- The intensity of the AE will be described in terms of mild, moderate or severe according to the investigator's clinical judgment. The intensity must be independent of the assessment of the seriousness of the AE.

Mild: Transient symptoms, no interference with the subject's daily activities, acceptable.

Moderate: Marked symptoms, moderate interference with the subject's daily activities but still acceptable.

Severe: Considerable interference with the subject's daily activities, unacceptable.

- The duration of the event will be described by the start date and end date.
- The causal relationship of the event to Tysabri® will be described in terms of:

Related: Event is related to Tysabri®. Related AEs or SAEs are classified as adverse reaction (AR) or serious adverse reaction (SAR).

Probable: Good reason and sufficient documentation to assume a causal relationship between Tysabri® and the AE. Probable related AEs or SAEs are classified as adverse reaction AR or SAR.

Possible: A causal relationship is likely and cannot be excluded. Possible related AEs or SAEs are classified as AR or SAR.

Unlikely: The event is most likely related to aetiology other than Tysabri® .

Unknown: Impossible to assess e.g. because of insufficient evidence, conflicting data or poor documentation.

Not related: No relationship to Tysabri®.

- The outcome of the event will be described in terms of:

Recovered / resolved

Recovering / resolving

Not recovered / not resolved

Recovered / resolved with sequelae

Fatal

Unknown

Determination of expectedness will be based on the contents of the SmPC in each participating country.

Note: Insofar as possible all AEs should be followed-up to determine the final outcome of the event. Details of follow-up should be given (e.g. if specific treatment is required or if hospitalisation is required).

11.3 Time Period, and Frequency of Detecting AEs and SAEs

From the time a patient consents to participate in the trial until he/she has completed the trial (without any follow up period), all AEs will be reported in the CRF.

All SAEs assessed as related to trial participation (e.g. protocol-mandated procedures, invasive tests or change in existing therapy) will be reported promptly to aCROnordic A/S. SAEs will be reported by filling out and sending the SAE form within 24 hours to aCROnordic A/S once the investigator determines that the event meets the protocol definition of an SAE. aCROnordic A/S will forward the information to Biogen A/S which will process the SAEs and when relevant notify the regulatory authorities and/or IEC.

SAEs occurring after trial termination must be reported if considered related to the treatment during the trial.

After the initial SAE report the investigator is required, proactively, to provide further information regarding the patient's condition. All follow-up information must be forwarded to aCROnordic A/S as it becomes available.

The investigator must follow-up all patients with SAEs until the event has subsided (or disappeared), the condition has stabilised, the event is otherwise explained or the patient is lost to follow-up.

For all deaths reported at any time, available autopsy reports and relevant medical reports should be faxed to aCROnordic A/S.

Information about a suspected unexpected serious adverse reaction (SUSAR) which occurs during the course of the trial and is fatal or life-threatening will be reported by Biogen Idec A/S as soon as possible to the competent authorities of all European Economic Area (EEA) States in which the trial is being conducted and the relevant ethics committee. This will be done no later than 7 days after the Biogen Idec A/S first became aware of the reaction. Any additional relevant information should be sent within 8 days of the initial report. A SUSAR, which is not fatal or life-threatening will be reported by Biogen Idec A/S as soon as possible, and no later than 15 days after becoming aware of the reaction to the competent authorities of all EEA States in which the trial is being conducted and the relevant ethics committee.

11.4 Pregnancies and Breast Feeding

Subjects who become pregnant during the trial must be withdrawn. The subject will be asked to attend follow-up visits and the outcome of the pregnancy will be followed including reporting on the state of the child 8 to 12 weeks after delivery. The investigator will be asked to fill in a pregnancy form.

Patients receiving treatment with Tysabri® shall not breast feed their children.

See national SmPC for Tysabri® in each participating country for more details.

12 Data Analysis and Statistical Considerations

12.1 Hypotheses

In general, the null hypothesis is that there is no change in the endpoint measurements at baseline compared to a later time-point measurement in the trial (for the respective variable being compared), against the alternative of some difference.

12.2 Sample Size Considerations

In order to achieve a power of 90 % to detect 25 % improvement in fatigue when performing a Student's t-test a total of 168 events should be included in the trial. It is assumed that the ratio between standard deviation (SD) and mean is close to 1.0.

A total of 200 subjects will be screened in the trial to include approximately 168 subjects in the trial thus compensating for screening failures, withdrawn or early terminated subjects.

12.3 Data Analysis Considerations

12.3.1 Analysis Data Sets and Populations

All subjects enrolled and treated with Tysabri® will constitute the intention to treat (ITT) analysis set.

A per-protocol (PP) analysis set will consist of all subjects in the ITT analysis excluding those that did have major protocol violations (e.g. not fulfilling inclusion and exclusion criteria) do not complete the trial or have missing measurement of the primary endpoint at baseline and 12 month after baseline.

12.3.2 Treatment Comparisons

12.3.2.1 Primary Comparisons of Interest

The primary variable is the measurement of fatigue (FSMC) at baseline (month 0) compared to the measurement 12 months after baseline.

12.3.2.2 Other Comparisons of Interest

Secondary variables and comparisons are measurement at baseline compared to measurement at month 6 and 12 after baseline of the following variables:

- FSMC (including measurement at month 3 and 9)
- Capacity for work (CWQ)
- HRQoL (SF-12)
- Sleepiness (ESS)
- Depression (CES-D)
- Cognitive impairment (SDMT done before 6MWT and PASAT)
- Physical activity induced exhaustion (BS-CR10 done before 6MWT)
- Speed of walking (6MWT)

- Status of MS disease progression (EDSS)
- Amount of walking (step counter)

Furthermore, fatigue at baseline and after 12 months of treatment with Tysabri® in subjects with different pre-treatment background (naïve, glaitiramer acetate, IFN-β) is also compared.

SDMT and BS-CR10 is done twice at baseline (0 month), 6 months and 12 months. At each of the 3 visits are tests performed before 6MWT compared with tests performed after 6MWT (e.g. SDMT done before 6MWT are compared to SDMT done after 6MWT at three occasions during the trial).

12.3.3 Interim Analysis

No interim analysis is planned for this trial.

12.3.4 Key Elements of Analysis Plan

All statistical testing will be done as 2-sided on a 5 % level of significance, and in particular all confidence intervals (CI) will be 95 % intervals.

In general, no specific procedure will be done for treating missing data and testing for multiplicity will not be considered.

12.3.4.1 Efficacy Analyses

All analysis on the primary as well as the secondary efficacy variables will be based on the change from baseline measurements, that is, the subjects' difference in measurement from baseline to the later trial visit. It will be addressed in the statistical analysis plan (SAP) how the various efficacy variables are evaluated from the respective questionnaires.

All efficacy endpoints will be summary tabulated with number (*N*) of non-missing observations, mean, SD, median, min and max. For categorical data, descriptive statistics will be presented with total number of observations (exposed subjects), number (*N*) with percentage of observations in the various categories of the endpoint, where percentage will be based on the total number of observations (which include eventual missing values).

Tabulation will be done by visit number (normally in columns) in case of repeated measurements. Unless otherwise stated, subject listings corresponding to the specified tables will be provided.

An appendix to the statistical analysis plan (SAP) will outline the tables and listings to be presented. Also, it will be addressed in the SAP how the various variables are evaluated from the respective questionnaires.

Further, a correlation analysis between the primary endpoint (based on FSMC scoring) and some of the secondary endpoints will be carried out to investigate any eventual interrelation between the respective variables. The following secondary variables will be considered in the correlation analysis:

- SDMT

- PASAT
- BS-CR10
- 6MWT
- CES-D
- EDSS
- Step counter

All efficacy variables will be analysed in a repeated measurement model including time as a categorical fixed effect with levels corresponding to the trial visits. Centres will be included as random effect, if significant. For continuous variables, change from baseline values will be used as response and baseline measurement of the variable will also be included as a fixed effects. Variables representing numbers (e.g. PASAT) will be treated in a more general linear mixed model also including the baseline value as response if change from baseline value is not considered appropriate. The model will use an appropriate link function, for instance the logarithm in a Poisson model set-up. Further, in the models analysing the SDMT and BS-CR10 scores, an additional variable will be included (fixed effect) to account for whether the test is performed before or after the 6MWT, such that a pair-wise comparison can be estimated for the two tests (before and after).
Least-square means will be presented at each visit with the corresponding 95 % CI and *p*-value for testing the null hypothesis of the mean value equal to 0. Similar estimates will be presented at the respective visits, for comparing each of the SDMT and BS-CR10 scores before and after the performance of the 6MWT.

In case that severe skewness of data is observed for an endpoint, an alternative analysis based on the non-parametric Wilcoxon signed rank test for analysing the median of the response, will be considered. Further, the associated Hodges-Lehmann CI for the median difference will be supplemented.

Individual data for the primary endpoint will be plotted against each of the above variables and for each time of measurement, in separate (scatter) plots.

12.3.4.2 Safety Analyses

All AEs will be medical dictionary for regulatory activities (MedDRA) coded by body system, preferred term, number of events and subjects experiencing the respective events will be tabulated by the MedDRA codes. A separate table for the SAEs will be done unless a very few events occurs. All AEs as well as SAEs will be subject listed, separately.

Concomitant medications and medical history will only be subject listed.

13 Trial Administration

13.1 Regulatory and Ethical Considerations, Including the Informed Consent Process

aCROnordic A/S or aCROnordic A/S in cooperation with other contract research organisations (CROs) will obtain favourable opinion/approval to conduct the trial from the appropriate regulatory agency in accordance with any applicable country-specific regulatory requirements prior to a site initiating the trial in that country.

The trial will be conducted in accordance with all applicable regulatory requirements.

The trial will also be conducted in accordance with GCP, all applicable subject privacy requirements and the guiding principles of the Declaration of Helsinki. This includes but is not limited to, the following:

- IEC review and favourable opinion/approval to conduct the trial and of any subsequent relevant amended documents
- Subject ICF
- Investigator reporting requirements

Biogen Idec A/S will provide full details of the above either verbally, in writing or both.
Written informed consent will be obtained for each subject before he or she can participate in the trial.

13.2 Definition of Source Data

The following questionnaires used in the trial are source data: FSMC, CWQ, SF-12, ESS, CES-D, SDMT and EDSS. The results from other questionnaires should be entered directly in the CRF. All other documentation in the CRF must be documented primarily in the subject's medical records, except for ethnic origin.

13.3 Direct Access

Direct access to all source data and documents will be mandatory for representatives from Biogen Idec A/S, CROs, ethics committees and regulatory authorities and other national authorities (e.g. data protection agency).

If an audit or inspection occurs, the investigator and institution agree to allow the auditor/inspector direct access to all relevant documents and to allocate his/her time and the time of his/her staff to the auditor/inspector to discuss findings and any relevant issues.

13.4 Quality Control (Trial Monitoring)

In accordance with applicable regulations, GCP, and BiogenIdec A/S procedures, monitors will contact the site prior to the start of the trial to review with the site staff the protocol, trial requirements and their responsibilities to satisfy regulatory, ethical and Biogen Idec A/S requirements. When reviewing data collection procedures, the discussion will also include identification, agreement and documentation of data items for which the CRF will serve as the source document.

The trial will be monitored consistent with the demands of the trial and site activity to verify that the:

- Data are authentic, accurate, and complete
- Safety and rights of subjects are being protected
- Trial is conducted in accordance with the currently approved protocol and any other trial agreements, GCP and all applicable regulatory requirements

The investigator and the head of the medical institution (where applicable) agrees to allow the monitor direct access to all relevant documents.

13.5 Quality Assurance

To ensure compliance with GCP and all applicable regulatory requirements, BiogenIdec A/S may conduct a quality assurance audit. Regulatory agencies may also conduct a regulatory inspection of this trial. Such audits/inspections can occur at any time during or after completion of the trial. If an audit or inspection occurs, the investigator and institution agree to allow the auditor/inspector direct access to all relevant documents and to allocate his/her time and the time of his/her staff to the auditor/inspector to discuss findings and any relevant issues.

13.6 Trial and Site Closure

Upon completion or premature discontinuation of the trial, the monitor will conduct site closure activities with the investigator or site staff, as appropriate, in accordance with applicable regulations, GCP and BiogenIdec A/S procedures.

In addition, Biogen Idec A/S reserves the right to temporarily suspend or prematurely discontinue this trial at any time for reasons including but not limited to safety or ethical issues or severe non-compliance. For multi-centre studies, this can occur at one or more or at all sites. If Biogen Idec A/S determines such action is needed, Biogen Idec A/S will discuss this with the investigator or the head of the medical institution (where applicable), including the reasons for taking such action at that time. When feasible, Biogen Idec A/S will provide advance notification to the investigator or the head of the medical institution, where applicable, of the impending action prior to it taking effect.

Biogen Idec A/S will promptly inform all other investigators or the head of the medical institution (where applicable) and/or institutions conducting the trial if the trial is suspended or terminated for safety reasons. Biogen Idec A/S will also promptly inform the regulatory authorities of the suspension or termination of the trial and the reason(s) for the action. If required by applicable regulations, the investigator or the head of the medical institution (where applicable) must inform the IEC promptly and provide the reason for the suspension or termination.

13.7 Records Retention

Following closure of the trial, the investigator or the head of the medical institution (where applicable) must maintain all site trial records, except for those required by local regulations to be maintained by someone else, in a safe and secure location. The records must be maintained to allow easy and timely retrieval, when needed (e.g. audit or inspection), and, whenever feasible, to allow any subsequent review of data in conjunction with assessment of the facility, supporting systems and staff. Where permitted by local laws/regulations or institutional policy, some or all of these records can be maintained in a format other than hard copy (e.g. microfiche, scanned and electronic); however, caution needs to be exercised before such action is taken. The investigator must assure that all reproductions are legible and are a true and accurate copy of the original and meet accessibility and retrieval standards, including re-generating a hard copy, if required. Furthermore, the investigator must ensure there is an acceptable back-up of these reproductions and that an acceptable quality control process exists for making these reproductions.

Biogen Idec A/S will inform the investigator of the time period for retaining these records to comply with all applicable regulatory requirements. The minimum retention time will

meet the strictest standard applicable to that site for the trial, as dictated by any institutional requirements or local laws or regulations, or Biogen Idec A/S standards/procedures; otherwise, the retention period will default to 15 years.

The investigator must notify Biogen Idec A/S of any changes in the archival arrangements, including, but not limited to, the following: archival at an off-site facility, transfer of ownership of the records in the event the investigator leaves the site.

13.8 Provision of Trial Results and Information to Investigators

When required by applicable regulations, the investigator signatory for the clinical trial report will be determined at the time the report is written. When the clinical trial report is completed Biogen Idec A/S will provide the investigator with a full summary of the trial results. The investigator is encouraged to share the summary results with the subjects, as appropriate. In addition, the investigator will be given reasonable access to review the relevant statistical tables, figures and reports and will be able to review the results for the entire trial at a Biogen Idec A/S site or other mutually agreeable location.

13.9 Data Management

The data collection tool for this trial will be paper CRF. Subject personal data will never be collected by nor transmitted to aCROnordic A/S. Subject data necessary for analyses and reporting will be entered/transmitted into a validated database or data system. Clinical data management will be performed in accordance with applicable aCROnordic A/S standards and data cleaning procedures. Original CRFs will be retained by aCROnordic A/S while the investigator will retain a copy.

13.10 End of Trial Definition

The "end of trial" is defined by the completion of the last visit of the last trial subject in any participating centre.

13.11 Finance and Insurance

All agreements between the Investigator and CROs (on behalf of Biogen Idec A/S) must be signed prior to inclusion of the first patient in the clinical trial. The agreement must clearly state the rights and obligations of the parties concerned and include a detailed financial settlement.

Every patient participating in the trial will be insured in accordance with the local legal requirements against trial related injuries to health which may occur during the trial.

Excluded from this however are injuries to health and deteriorations of illnesses already in existence which would have occurred or continued to exist even if the patient had not taken part in the clinical trial.

The insurance cover is jeopardised if the patient fails to report immediately to the investigator or responsible physician any injury to health which might have resulted from participation in the clinical trial, or if the patient undergoes any other medical treatment

without their consent before the clinical trial has been completely finished - insofar as the individual patient is concerned.

Any injury to health, which might have occurred as a result of participation in the clinical trial, must be reported by the patient to the insurer without delay. The investigator is obliged to make such a report in any case.

The patient insurance will be arranged by Biogen Idec A/S on the basis of the final version of the trial protocol.

13.12 Publication Policy

An integrated clinical trial report will be prepared by aCROnordic A/S and reviewed by Biogen Idec A/S. BiogenIdec A/S will send the summary of the clinical trial report (according to CPMP/ICH/137/95) to the regulatory authority and IEC no later than 90 days after the end of the trial.

No data from the clinical trial may be published, presented or communicated, except to regulatory authorities, prior to the release of the internal clinical trial report, unless approved by Biogen Idec A/S in writing. The investigator agrees not to discuss externally or publish any result from the trial without the possibility of Biogen Idec A/S to give comments for up to 60 days after receipt of the manuscript.

In the event of a publication, the names of the authors and their order of appearance will most likely be as follows: Coordinating investigator, other investigators (according to number of patients included and time of inclusion), then representatives of Biogen Idec A/S and aCROnordic A/S.

All persons designated as authors should qualify for authorship and all those who qualify should be listed.

Each author should have participated sufficiently in the work to take public responsibility or appropriate portions of the content.

Authorship credit should be based on (1) substantial contributions to conception and design, acquisition of data or analysis and interpretation of data; (2) drafting the article or revising it critically for important intellectual content; (3) final approval of the version to be published. Authors should meet conditions 1, 2 and 3.

If the results from the trial are not intended to be published in any scientific journal then it is the responsibility of BiogenIdec A/S to communicate both positive and negative results to the community by publishing a summary of the results within 2 years after the end of trial in a communication report posted at BiogenIdec A/S website or in other related media.

14 References

(1) ICH Harmonised Tripartite Guideline for Good Clinical Practice with EC 2001/20/EC & GCP Directive. Third 2005 ed. Informa UK Ltd; 1996.

(2) Krupp L. Fatigue is intrinsic to multiple sclerosis (MS) and is the most commonly reported symptom of the disease. Mult Scler 2006 Aug;12(4):367-8.

(3) Lerdal A, Celius EG, Krupp L, Dahl AA. A prospective study of patterns of fatigue in multiple sclerosis. Eur J Neurol 2007 Dec;14(12):1338-43.

(4) Putzki N, Katsarava Z, Vago S, Diener HC, Limmroth V. Prevalence and severity of multiple-sclerosis-associated fatigue in treated and untreated patients. Eur Neurol 2008;59(3-4):136-42.

(5) Fatigue and Multiple Sclerosis : Evidence-Based Management Strategies for Fatigue in Multiple Sclerosis. 1998 Oct.

(6) Penner IK, Bechtel N, Raselli C, Stocklin M, Opwis K, Kappos L, et al. Fatigue in multiple sclerosis: relation to depression, physical impairment, personality and action control. Mult Scler 2007 Nov;13(9):1161-7.

(7) Penner IK, Raselli C, Stoecklin M, Opwis L, Kappos L. The FSMC (Fatigue Scale for Motor and Cognitive Functions) - a new instrument to assess MS-related fatigue. Neuropsychology 2007 Oct 13; Poster topic 20.

(8) Rudick RA, Stuart WH, Calabresi PA, Confavreux C, Galetta SL, Radue EW, et al. Natalizumab plus interferon beta-1a for relapsing multiple sclerosis. N Engl J Med 2006 Mar 2;354(9):911-23.

(9) Polman CH, O'Connor PW, Havrdova E, Hutchinson M, Kappos L, Miller DH, et al. A randomized, placebo-controlled trial of natalizumab for relapsing multiple sclerosis. N Engl J Med 2006 Mar 2;354(9):899-910.

(10) Putzki N, Kollia K, Katsarava Z, Woods S, Igwe E, Diener H. Multiple sclerosis related fatigue improves after treatment initiation with natalizumab. Neuropsychology 2007 Oct 13;Poster topic 20.

(11) Statistical Principles for Clinical Trials. ICH Topic E 9. CPMP/ICH/363/96. 1998.

(12) Quality Metric website - SF-12. internet 2008 October 21 Available from: URL: www.qualitymetric.com

(13) Radloff LS. The CES-D scale: A self-report depression scale for research in the general population. Applied Psycological Measurement 2008;1:385-401.

(14) Gronvall D.M. Paced auditory serial-additon task: a measure of recovery from concussion. Percept Mot Skills 2008;44(2):367-73.

(15) Rao SM, Leo GJ, Haughton VM, St Aubin-Faubert P, Bernardin L. Correlation of magnetic resonance imaging with neuropsychological testing in multiple sclerosis. Neurology 1989 Feb;39(2 Pt 1):161-6.

(16) Smith A. Symbol Digit Modalities Test Manual. Western Psycological Services; 2002.

(17) ATS statement: guidelines for the six-minute walk test. Am J Respir Crit Care Med 2002 Jul 1;166(1):111-7.

(18) Goldman MD, Marrie RA, Cohen JA. Evaluation of the six-minute walk in multiple sclerosis subjects. Multiple Sclerosis 2008;14:383-90.

(19) Guyatt GH, Sullivan MJ, Thompson PJ, Fallen EL, Pugsley SO, Taylor DW, et al. The 6-minute walk: a new measure of exercise capacity in patients with chronic heart failure. Can Med Assoc J 1985 Apr 15;132(8):919-23.

(20) Kappos L. Neurostaus - Standardised Neurological Examination and Assessment of Kurtzke's *Functional Systems and Expanded Disability Status Scale* (slighty modified from J.F. Kurtzke, Neurology 1983;33:1444-1452): Department of Neurology, University Hospitals, CH-4031 Basel, Version 12/2004. 2004 Dec.

(21) Kurtzke JF. Rating neurologic impairment in multiple sclerosis: an expanded disability status scale (EDSS). Neurology 1983 Nov;33(11):1444-52.

(22) Focht BC, Sanders WM, Brubaker PH, Rejeski WJ. Initial Validation of CSA Activity Monitor During Rehabilitative Exercise Among Older Adults With Chronic Disease. Journal of Aging and Physical Activity 2003;3:293-304.

(23) Polman CH, Reingold SC, Edan G, Filippi M, Hartung HP, Kappos L, et al. Diagnostic criteria for multiple sclerosis: 2005 revisions to the "McDonald Criteria". Ann Neurol 2005 Dec;58(6):840-6.
